# Seasonal respiratory virus circulation was diminished during the COVID‐19 pandemic

**DOI:** 10.1111/irv.13065

**Published:** 2022-11-11

**Authors:** Aliisa Heiskanen, Yannick Galipeau, Julian Little, Leanne Mortimer, Karamchand Ramotar, Marc‐André Langlois, Curtis L. Cooper

**Affiliations:** ^1^ School of Epidemiology and Public Health, Faculty of Medicine University of Ottawa Ottawa Canada; ^2^ Department of Biochemistry, Microbiology & Immunology, Faculty of Medicine University of Ottawa Ottawa Canada; ^3^ Eastern Ontario Regional Laboratory Association Ottawa Canada; ^4^ University of Ottawa Centre for Infection, Immunity and Inflammation (CI3) Ottawa Canada; ^5^ Ottawa Hospital Research Institute Ottawa Canada

**Keywords:** COVID‐19, influenza, SARS‐CoV‐2, seasonal respiratory viruses

## Abstract

**Background:**

Measures introduced during the COVID‐19 pandemic intended to address the spread of SARS‐CoV‐2 may also influence the incidence of other common seasonal respiratory viruses (SRV). This evaluation reports laboratory‐confirmed cases of common SRV in a well‐defined region of central Canada to address this issue.

**Methods:**

Surveillance data for common non‐SARS‐CoV‐2 SRV in Ottawa, Canada, was provided by the Eastern Ontario Regional Laboratory Association (EORLA) reference virology lab. Weekly reports of the number of positive tests and the proportion that yielded positive results were analyzed from August 26, 2018, to January 2, 2022.

**Results:**

A drastic reduction in influenza and other common SRV was observed during the 2020–2021 influenza season in the Ottawa region. Influenza was virtually undetected post‐SARS‐CoV‐2 emergence. Rhinoviruses and enteroviruses were the only viruses that remained relatively unaffected during this period.

**Conclusions:**

We speculated that the introduction of nonpharmaceutical measures including masking to prevent SARS‐CoV‐2 transmission contributed to the near absence of SRV in the Ottawa region. These measures should remain a key component in addressing spikes in SRV activity and future pandemics.

## 1 INTRODUCTION

Since emerging in late 2019, SARS‐CoV‐2 has caused unprecedented morbidity and mortality worldwide. Public health policymakers have implemented nonpharmaceutical public health measures to mitigate SARS‐CoV‐2 spread including the use of face coverings, targeted lockdowns and stay‐at‐home orders, physical distancing, school closures, restricted international travel, mandated quarantine, and reduced close contacts. In Ontario, most of these measures were put into place from March 12 to 30, 2020. Testing regulations and access to testing varied by stage of the pandemic and availability of tests.

Influenza, RSV, and other common respiratory viruses typically follow predictable seasonal patterns with high activity levels in the winter months. Australia and New Zealand saw a 98% decrease in influenza incidence during the 2020 peak influenza or “flu” season. Reduced activity was also reported for common respiratory illnesses such as the respiratory syncytial virus (RSV); seasonal human coronaviruses (HCoV); parainfluenza virus types 1, 2, 3, and 4 (PIVs); and human metapneumovirus (HMPV). Irregular seasonal behavior was observed in enteroviruses (EV), adenoviruses (AV), and rhinoviruses (RV) throughout the Southern Hemisphere.^1^, ^2^ Similar patterns were observed throughout the winter influenza season in the Northern Hemisphere. Minimal influenza and RSV activity were reported during peak seasons in the UK and Europe, where most respiratory illness‐related healthcare visits were accredited to COVID‐19.^3^ Surveillance data from the United States showed that influenza and RSV were circulating at historically low levels from March 2020 to May 2021. HCoV, PIV, and HMPV activity decreased in March 2020 and remained low through May 2021, after which HCoV and PIV rose to prepandemic levels. The increase in HCoV, RSV, and PIV in the spring of 2021 was inconsistent with historical seasonal peaks usually observed in the winter months. RV and EV activity decreased in March 2020 but returned to prepandemic levels in May 2020.^4^ A study utilizing Canadian surveillance data reported an absence of most respiratory viruses including influenza A, influenza B, and RSV during 2020–2021. The exception was enteroviruses and rhinoviruses, which were continuously detected throughout the pandemic.^5^

This evaluation utilized surveillance data to evaluate the effect of SARS‐CoV‐2 pandemic countermeasures on seasonal respiratory virus circulation in the Ottawa region. Ottawa is a diverse city consisting of a broad spectrum of communities and socioeconomic conditions. Consequently, we believe that our results are broadly applicable to developed world settings.

## 2 METHODS

### 2.1 Design and setting

This observational study was conducted using publicly available surveillance data from the Ottawa region. The catchment area included the 1.3 million people living in the Champlain region, Ontario's most eastern Local Health Integrated Network (LHIN). The Champlain region population is concentrated in the city of Ottawa and also spans east and west sharing a 465 km border with Quebec. This region is ethnically and linguistically diverse; 19.8% identify French as their first language, and at least 2.8% are recent immigrants.^6^ The vast majority of samples were obtained from patients assessed at The Ottawa Hospital and Children's Hospital of Eastern Ontario (CHEO) thereby capturing both adult and pediatric population data.

### 2.2 Data sources

Specimens were collected from the Champlain region population and submitted to the Eastern Ontario Regional Laboratory Association (EORLA) regional reference virology lab based at CHEO. These data are in the public domain and circulated weekly to several sources including the Public Health Agency of Canada (PHAC) as part of the Respiratory Virus Detection Surveillance System and FluWatch report.^7^ All data included in this analysis were obtained from publicly available de‐identified datasets. As such, ethics board and laboratory approval were not required.

### 2.3 Measures/variables

Respiratory viruses of interest included: influenza A, influenza B, respiratory syncytial virus, parainfluenza viruses 1–4, human metapneumovirus, seasonal human coronaviruses (HCoV‐229E, HCoV‐OC42, HCoV‐NL63, and HCoV‐HKU1), adenovirus, enterovirus, rhinovirus, and SARS‐CoV‐2. Health Canada‐approved assays were used for viral detection. Almost all tests were multiplex reverse transcriptase real‐time PCR assays, except for one SARS‐CoV‐2 assay that was a TMA‐based nucleic acid amplification assay. A description of the tests used for each virus can be found in Table 1. Data from non‐SARS‐CoV‐2 respiratory virus activities were reported weekly from August 26, 2018, to December 26, 2021. SARS‐CoV‐2 data were reported from August 26, 2020, to December 26, 2021. Influenza season was defined as from the beginning of November to the end of April. A case was defined as a laboratory‐confirmed positive test for each respiratory virus. Percent positive values were calculated as the number of tests that yielded positive results divided by the total number of tests performed for a given week expressed as a percentage for each virus.

**TABLE 1:** Types of tests used to measure the seroprevalence of each respiratory virus. Location of where testing was performed within the catchment area and contextual factors and restrictions in place that may have impacted the measured seroprevalence of SARS‐CoV‐2 and common respiratory viruses in the Ottawa region.

Virus	Type of test used	Location	Testing restrictions
Influenza A	‐Hologic FluA/B/RSV assay ‐Luminex Aries FluA/B/RSV assay	‐Testing performed for some of the community hospitals (QCH, Montfort, Glengarry, Cornwall, Hawkesbury, Renfrew) in addition to TOH and CHEO	‐Largely unrestricted. Testing was almost always performed if it is requested.
Influenza B			
RSV			
HMPV	‐Allplex respiratory virus panel 2 ‐Allplex respiratory virus panel 3	‐>99% of requests are from TOH and CHEO	‐Mid 2020 onward, most hospitalized patients were not tested for respiratory viruses other than influenza A, influenza B, RSV, SARS‐CoV‐2 ‐Testing was restricted and must be approved by a microbiologist unless ordered by ID
Endemic HCOVs			
Enterovirus			
Rhinovirus			
Adenovirus			
Parainfluenza viruses (1–4)			
SARS‐CoV‐2	‐Allplex 2019‐n CoV assay ‐Cobas SARS‐CoV‐2 assay ‐Aptima SARS‐CoV‐2 assay ‐Fusion SARS‐CoV‐2 assay	‐Entire catchment area	‐Subject to local and provincial guidelines

Abbreviations: CHEO, Children's Hospital of Eastern Ontario; HCoV, human coronavirus; HMPV, human metapneumovirus; QHC, Queensway Carleton Hospital; RSV, respiratory syncytial virus; SARS‐CoV‐2, severe acute respiratory syndrome coronavirus 2; TOH, The Ottawa Hospital.

### 2.4 Statistical methods

The number of positive laboratory results and the percent positive value of each virus were summarized using combination line and bar graphs. Enterovirus and rhinovirus data were reported as a single value. Data for parainfluenza virus types 1–4 were combined and presented in one chart due to the trivial number of reported cases.

## 3 RESULTS

**FIGURE 1:**
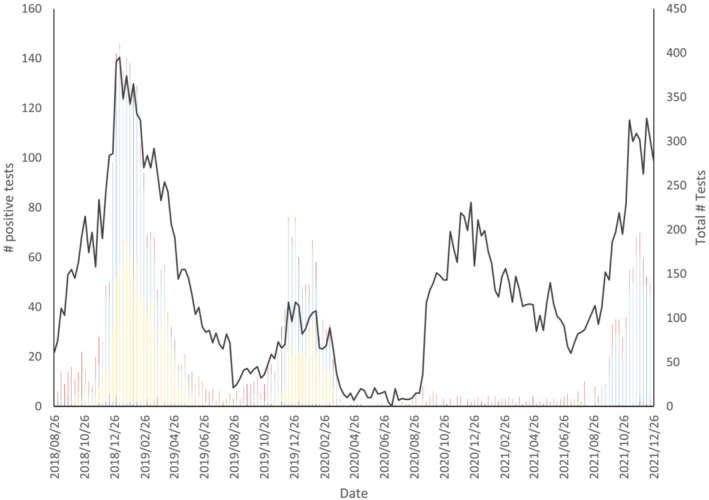
Total number of tests performed and number of positive common respiratory virus cases collected weekly in the Ottawa region. Legend: 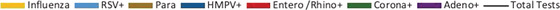

An overview of the number of non‐SARS‐CoV‐2 specimens submitted to the EORLA laboratory for testing and the number of positive results for each virus from 2018 to 2021 is provided in Figure 1. From the beginning of November to the end of April, approximately 7300 specimens were tested in the 2018–2019 season, 1800 in 2019–2020, and 9800 in 2020–2021. Viral peaks largely consisting of influenza and RSV occurred from November to February of the 2018–2019 and 2019–2020 winter season. An obvious absence of viral respiratory illness was reported from March 2020 onward including during the typical flu season of 2020–2021. Enterovirus and rhinovirus returned to endemic levels in August 2020, whereas viral activity for other respiratory illnesses remained low. RSV resumed prepandemic activity levels in September 2021.

**FIGURE 2:**
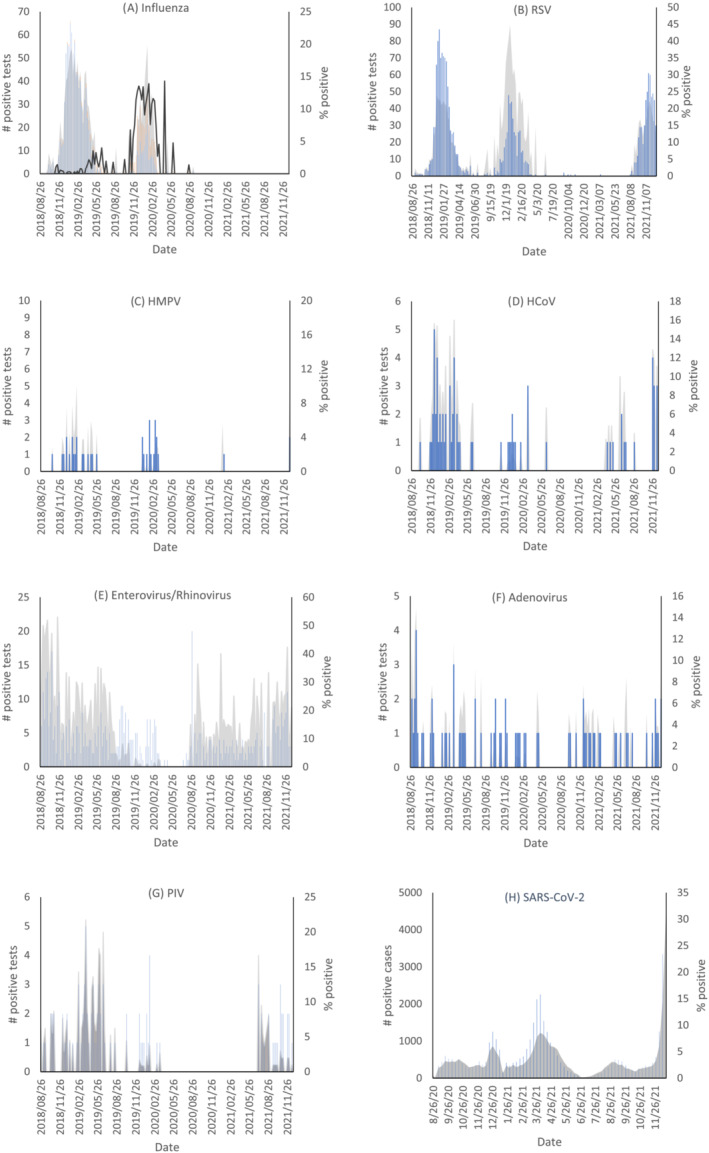
Number of positive tests and proportion of submitted tests that yielded positive results for influenza A and B, respiratory syncytial virus, human metapneumovirus, endemic human coronaviruses, entero/rhinoviruses, adenoviruses, parainfluenzas 1–4, and COVID‐19 collected weekly from the Ottawa region. The shaded regions and line (2a) represent the test positivity as a percent. The bars represent the number of positive test results reported each week. Legend: 2a) 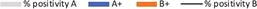; 2b) 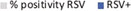; 2c) 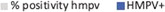; 2d) 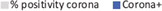; 2e) 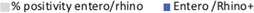; 2f) 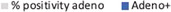; 2g) 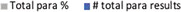; 2h) 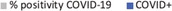

The number of positive specimens and the percent of specimens that tested positive for each respiratory virus are described in Figure 2. Influenza A was dominant in the 2018–2019 winter season and both influenza A and B were prominent in the winter of 2019–2020. Influenza activity was negligible following the onset of the COVID‐19 pandemic in March 2020 (Figure 2a). The RSV season in Ontario and North America in general usually starts between November and January and generally lasts 3–4 months.^8^ Besides an absence of activity in 2020–2021, standard RSV seasonality was observed throughout the study period (Figure 2b). HMPV was observed at consistently low levels from December 2018 to June 2019 and December 2019 to February 2020. No activity was observed in March 2020 and one spike in detection was recorded in February 2021 (Figure 2c). Typical endemic coronavirus seasonality consistent with pre‐COVID‐19 seasonal norms occurred in the 2018–2019 season with a peak from December to February and continued activity into early spring of 2019. Reduced incidence was detected the following winter with few positive cases and no activity identified over the 2020–2021 winter. Following several atypically early cases of HCoV in the spring of 2021, HCoV began to increase to prepandemic levels in the winter of 2021–2022 (Figure 2d). Enterovirus and rhinovirus activity remained largely unchanged by the COVID‐19 pandemic aside from a brief period from March 2020 to July 2020, in which few cases were detected. A spike of positive specimens in August 2020 preceded a return to prepandemic levels of viral activity (Figure 2e). Two positive cases of adenovirus were detected from March 2020 to September 2020, after which approximately one case was found every few weeks, analogous to prepandemic incidence rates (Figure 2f). In 2018–2019, parainfluenza activity was highest from February to June. Reduced activity was seen in the 2019–2020 season, with no positive cases detected in the peak 2020–2021 winter season. Parainfluenza activity returned to prepandemic levels in June of 2021 (Figure 2g). The reduction in common respiratory virus activity coincided with an increase in SARS‐CoV‐2 activity that began in August 2020 (Figure 2h).

## 4 DISCUSSION

A clear absence of surveyed non‐SARS‐CoV‐2 activity was observed beginning in March 2020, which lasted until August 2021. This timeline coincides with the introduction of public health measures in the Ottawa region and suggests that these measures implemented to mitigate SARS‐CoV‐2 transmission effectively reduced cases of seasonal respiratory viruses in the region. Consistent with trends observed worldwide,^2^, ^5^, ^9^, ^10^ influenza and RSV saw the greatest decline in activity. Influenza has been practically undetected in the region since the onset of the COVID‐19 pandemic. RSV was absent during the 2020–2021 season but returned to prepandemic levels the following season.

There are several reasons as to why COVID‐19 mitigation measures have proven to be highly effective against common respiratory viruses such as influenza compared to SARS‐CoV‐2. Although influenza and SARS‐CoV‐2 are both spread through droplet transmission, influenza has lower transmissibility (R_0_ = 1.3) compared to the ancestral strain of SARS‐CoV‐2 (R_0_ = 2.0–4.0).^11^ Consequently, measures including mask wearing and physical distancing may be more efficient at preventing influenza transmission.^4^ Further, a degree of immunity to common respiratory viruses is already present in the population. A higher dose exposure and persistent viral load may be required for common respiratory infections to flourish compared to the novel SARS‐CoV‐2, which may have a competitive advantage in an immunologically naïve population.^3^ In accordance with other jurisdictions, our evaluation also observed atypical HCoV and PIV activity. Children have been known to propagate respiratory illness to their households and communities, so it is possible that school closures may have blunted viral outbreaks that usually occur in the fall and winter.^4^, ^12^ Reopening of schools is likely a key explanation for the reappearance of RSV to prepandemic levels in the fall of 2021. Rhinoviruses, adenoviruses, and enteroviruses remained relatively unaffected by the COVID‐19 pandemic in this study and worldwide.^3^, ^4^, ^5^, ^9^ This persistence may be attributed to varied modes of transmission compared to SARS‐CoV‐2, higher survival time on surfaces, or lack of envelope, which could provide protection against sanitizing and handwashing behaviors.

Although there is compelling evidence that public health measures have reduced seasonal respiratory virus activity alternative hypotheses should be considered. Additional theories include the vast immunologic naivety of the host population and the capabilities of SARS‐CoV‐2 as a pathogenic competitor. Interferon‐stimulated immunity caused by a viral infection can provide nonspecific interference, deferring other viruses from establishing in a population.^13^, ^14^, ^15^ Interference is thought to limit coinfection rates and contributes to the seasonality of common respiratory viruses including influenza.^8^ It is possible that the presence of SARS‐CoV‐2 is outcompeting other viruses in the respiratory tract, delaying future outbreaks of respiratory viruses. Seasonal coronaviruses and SARS‐CoV‐2 share over 30% sequence homology in the S2 subunit, which may result in overlapping immune epitopes.^16^ Some studies have indicated that previous infection by seasonal HCoVs reduces the risk of infection and severity of SARS‐CoV‐2.^16^, ^17^, ^18^ Other studies have suggested that vaccination or natural infection by SARS‐CoV‐2 may boost antibodies and protect against endemic coronaviruses.^18^, ^19^, ^20^, ^21^, ^22^, ^23^, ^24^, ^25^ These views are not universal and conflicting findings argue that antibodies may be increased but do not provide protection from infection or hospitalization.^26^ Frequent recombination^27^, ^28^ and waning natural immunity^29^ characteristic of the HCoV family suggest that any cross‐immunity provided by infection or vaccination will not be strong enough to prevent future infections or inhibit seasonal HCoV circulation. Data from this study support the hypothesis that cocirculation of endemic coronaviruses and SARS‐CoV‐2 is possible, as seasonal HCoV activity returned to the Ottawa area in April 2021 despite ongoing waves of COVID‐19 infection. However, atypical seasonality was observed, which may reflect the effects of cross‐protection or viral interference. Further studies will hopefully fully elucidate the effect of SARS‐CoV‐2 on seasonal HCoV circulation and the complex immunological interactions between the two.

As measures are relaxed and vaccination is increasingly relied upon to control COVID‐19, the resurgence of respiratory viruses is expected and already occurring in some locations.^1^ Reduced transmission over the past 2 years has distorted the pool of susceptible individuals and may change the epidemiology of respiratory viruses for years to come. Early exposure and frequent stimulations early in life train the immune system to effectively respond to threats and have been linked to a fortified immune response in adulthood. Lack of immune stimulation caused by nonpharmaceutical public health measures over the past 2 years may result in immune deficits in children with negative consequences when the pandemic resolves.^30^ Opening international borders and increasing human mobility may also stimulate viral circulation as influenza transmission and seasonality have been partially attributed to international travel.^31^ Although rare, co‐infection by influenza and SARS‐CoV‐2 has been shown to result in an increased risk of severe illness and death (RR:5.92; 95%CI: 3.21–10.91) compared to patients with neither.^14^ Testing for influenza and other respiratory viruses should remain a priority. The COVID‐19 pandemic has demonstrated that nonpharmaceutical interventions are an important component in controlling viral transmission that should also be applied to seasonal respiratory virus outbreaks. Reinstating mask mandates or social distancing during influenza season or in high‐risk environments such as schools is anticipated to reduce transmission or even prevent future outbreaks of common respiratory viruses. The pandemic has also illustrated that remote work or school is feasible in some industries and supports social distancing theories. Now that the infrastructure is available, employees and students may be encouraged to stay home if they are feeling ill, reducing the risk of viral outbreaks in schools or workplaces. Clinicians should also be aware of an increase in respiratory virus activity that does not follow recurrent norms and encourage influenza vaccination.

This analysis is subject to several limitations. Due to the ecological nature of this study causation cannot be inferred. However, these findings are substantiated by a growing body of evidence that has documented similar patterns and suggest that public health measures meant to prevent SARS‐CoV‐2 transmission have interfered with seasonal respiratory virus activity worldwide.^2^, ^3^, ^4^, ^5^, ^9^, ^10^ It is possible that reduced testing and redirected patient flow underestimated viral activity. Those experiencing viral respiratory illness may have been sent to COVID‐19‐specific test sites instead of usual care where surveillance occurs. There was a short period during the summer of 2020 when testing using the extended respiratory virus panel (extended respiratory panel of all viruses excluding SARS‐CoV‐2, influenza A, influenza B, and RSV) was suspended to cope with COVID‐19. This may have resulted in an underestimation of the true number of cases. More detailed information on contextual factors that may have influenced the number of tests completed can be found in Table 1. Others may have been hesitant or incapable of accessing usual care or testing services amid lockdowns and COVID‐19 precautions. CHEO received 34% more specimens in the 2020–2021 influenza season compared to the 2018–2019 influenza season, so it is unlikely that testing capacity largely underestimated viral activity for influenza A, influenza B, or RSV. No seasonal spikes in influenza incidence, RSV hospital, and ICU admissions were observed, which also supports the proposal that findings truly represent reduced community circulation. Relatedly, Australia had more liberal testing criteria than Canada and still found only 33 positive influenza tests out of 60,031 tested specimens during peak season.^2^ Another limitation was that the influenza vaccination rate was not measured in this evaluation. Findings from the 2020–2021 Canadian Seasonal Influenza Vaccination Coverage Survey indicated that influenza vaccination coverage in the 2020–2021 season (40%) was slightly reduced but comparable to the 2019–2020 season (42%) and 2018–2019 season (42%).^32^ It is likely the rates were similar in this evaluation. However, as this variable was not measured, the exact proportion of the population vulnerable to infection is unknown and may have affected reported influenza infection rates. Finally, the surveillance data used in this analysis may not be fully representative of the Ottawa population at risk for RV infection as not all viral testing occurred at the CHEO virology laboratory. Individuals tested for SRVs through the Public Health Ontario Laboratory were not captured in these data.

## 5 CONCLUSION

This study describes a near absence of seasonal respiratory viruses in the Ottawa region for the 2020/2021 respiratory virus season. Findings from this study add to a growing body of evidence that suggests that public health measures introduced to prevent the spread of SARS‐CoV‐2 have also reduced laboratory‐confirmed seasonal respiratory virus infections. These measures should continue to be strategically utilized to prevent, or at least diminish, the burden of future seasonal respiratory virus spikes and in response to further pandemics.

## CONFLICTS OF INTEREST

The authors declare no conflicts of interest.

## ETHICAL APPROVAL

All data included in this analysis were obtained from publicly available de‐identified datasets; therefore, ethical approval or lab approval was not required.

## AUTHOR CONTRIBUTIONS

**Aliisa Heiskanen:** Conceptualization; data curation; formal analysis; investigation; methodology; project administration; writing‐original draft; writing‐review and editing. **Yannick Galipeau:** Methodology; writing‐review and editing. **Julian Little:** Writing‐review and editing. **Leanne Mortimer:** Data curation; methodology; resources; writing‐review and editing. **Karamchand Ramotar:** Data curation; methodology; resources; writing‐review and editing. **Marc‐André Langlois:** Writing‐review and editing. **Curtis L Cooper:** Conceptualization; investigation; methodology; project administration; supervision; writing‐review and editing.

### PEER REVIEW

The peer review history for this article is available at https://publons.com/publon/10.1111/irv.13065.

## Data Availability

The data that support the findings of this study are available on request from the corresponding author.
